# Facial asymmetry of the hard and soft tissues in skeletal Class I, II, and III patients

**DOI:** 10.1038/s41598-024-55107-4

**Published:** 2024-02-29

**Authors:** Jialing Li, Sujuan Wu, Li Mei, Juan Wen, Jamie Marra, Lang Lei, Huang Li

**Affiliations:** 1 Department of Orthodontics, Nanjing Stomatological Hospital, Affiliated Hospital of Medical School, Research Institute of Stomatology, Nanjing University, Nanjing, People’s Republic of China; 2 Department of Orthodontics, Nanjing Lishui Stomatological Hospital, Nanjing, People’s Republic of China; 3 Discipline of Orthodontics, Department of Oral Science, Faculty of Dentistry, University of Otago, Dunedin, New Zealand; 4 Department of Oral Diagnostic and Surgical Sciences, Faculty of Dentistry, University of Otago, Dunedin, New Zealand

**Keywords:** Facial asymmetry, Hard tissue, Soft tissue, CBCT, Orthodontics, Cone-beam computed tomography

## Abstract

To investigate and compare the facial asymmetry (hard and soft tissues) among skeletal Class I, II, and III patients. A total of 221 subjects, including skeletal Class I (n = 80), skeletal Class II (n = 75), and skeletal Class III (n = 66), were included in the study. CBCT, 22 skeletal landmarks, and 10 soft tissue landmarks were used for the measurements and the asymmetry index was calculated to assess the facial asymmetry. Statistical analyses included one-way ANOVA, Kruskal–Wallis test, and Spearman correlation analysis. The skeletal Class III patients presented greater asymmetry than Class II patients for 10 hard tissue landmarks and 3 soft tissue landmarks (p < 0.05). High correlation of asymmetry was found between four soft tissue landmarks and their corresponding skeletal landmarks (r_s_ ≥ 0.71), as well as Me and ANS (r > 0.86). The ANS and Me in 21.3% patients deviated to contralateral sides. The skeletal Class III patients had more facial asymmetry than the Class II patients. Soft tissues showed similar asymmetry as the underlying hard tissues rather than a compensation of the hard tissue asymmetry. The inconsistency in the deviation of Me and ANS may exacerbate facial asymmetry.

## Introduction

Facial symmetry is important for facial aesthetics; facial asymmetry, especially moderate and severe asymmetry, can negatively affect facial attractiveness, requiring orthodontic and orthognathic treatment^1^. The prevalence of facial asymmetry ranges from 11 to 37%^2,3^, and up to 50% when a more accurate and strict diagnostic method is used for the evaluation^4,5^. The etiology of facial asymmetry is considered to be multifactorial, including congenital, developmental, and environmental factors^1,6,7^.

A number of studies have evaluated the relationship between skeletal asymmetry and dental malocclusions. Most of these studies focused on a specific malocclusion^8–12^; some studies compared Class III with Class I^13–17^, Class II with Class I^18^, or Class III with Class II^19^. Few studies have directly compared facial asymmetry between Class I, II, and III malocclusions together^20,21^. Two previous studies on facial symmetry characteristics in subjects with different sagittal skeletal classes have suggested that the asymmetry was represented almost in the same way regardless of sagittal skeletal pattern; but their samples, including relative symmetry, moderate asymmetry, and severe asymmetry patients, were evenly distributed in each sagittal skeletal class^20,21^. Another study on the prevalence of facial asymmetry among three sagittal skeletal classes has reported that mandibular asymmetry was equally distributed among Class I, II, and III malocclusions^5^, however other studies also reported that facial asymmetry was more frequently related with Class III malocclusion^22,23^.

Soft tissue asymmetry is clinically important, especially with increasing aesthetic concerns^24^. Soft tissue morphology has been found to be highly correlated with the underlying skeletal structure^25^; however, soft tissue asymmetry has been observed even in the individual with symmetric hard tissues^26^. The relationship between the facial skeletal and soft tissue asymmetry is still under debate.

The aim of this study was to investigate facial asymmetry of the soft and hard tissues in subjects with skeletal Class I, II, and III relationships using three-dimensional (3D) cone-beam computed tomography (CBCT) images.

## Material and methods

### Participants

This study was designed following guidelines established in the Helsinki declaration and the STROBE statement. Sample size calculation was based on the literature^27^. Power analysis indicated that a sample size of 159 patients would provide an 80% probability of detecting a moderate effect of asymmetry index difference between different skeletal groups at a 95% confidence level using the ANOVA model (f = 0.25). A total of 221 normal subjects seeking dental treatment in the Stomatological Hospital of Nanjing University from 2019 to 2021 were included in the present study. All included subjects had CBCT images as a part of their records. Informed consent was obtained from all patients. Ethics of the study was approved by the Ethics Committee of the Nanjing Stomatological Hospital of Nanjing University (JX-2021-NL08).

The inclusion criteria were: (1) Adults (≥ 18 years old), with or without facial asymmetry; (2) Permanent dentition, without any missing teeth except third molars; (3) No facial deformity caused by congenital or pathological reasons, such as cleft lip and palate, tumor, cyst, temporomandibular joint disorder, or maxillofacial syndromes; and (4) Complete clinical records and CBCT data. The exclusion criteria were: (1) History of orthodontic or orthognathic treatment; (2) History of trauma to the face; and (3) Medical history of systemic disease.

Samples were divided into three groups based on the skeletal relationship^28,29^: skeletal Class I (0° < ANB < 5°), skeletal Class II (ANB ≥ 5°), and skeletal Class III (ANB ≤ 0°).

### Cone beam computed tomography (CBCT)

All CBCT images were acquired by NewTom VG or NewTom VGi (QR srl, Verona, Italy). Exposure conditions were 110 kV, 5 mA, 0.125-mm voxel size, 140 × 140 × 150 mm image size, and were performed according to the manufacturer's instructions. The sagittal plane of the patient's head was perpendicular to the ground; the orbito-auricular plane was parallel to the ground; the tongue was in the resting position; and the upper and lower jaws were in the cusp staggered position. Subjects were asked to keep their head steady, breathe calmly, not chew or swallow during filming.

All CBCT data were exported as Digital Imaging and Communications in Medicine (DICOM) files and reconstructed using the Materialise Interactive Medical Image Control System (Mimics Innovation Suite, Materialise, Belgium) for the measurements.

### Facial asymmetry assessment

**Figure 1:**
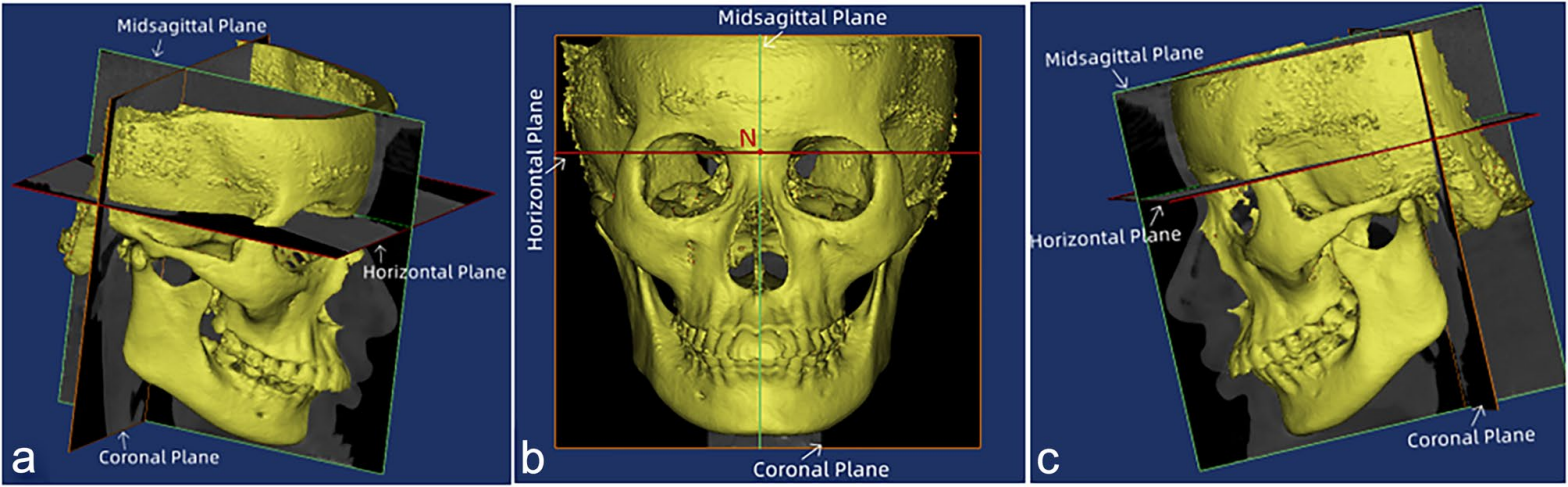
Schematic diagram of the 3D reference plane viewed from different positions (**a**) Right side view (**b**) Front view (**c**) Left side view.

**Figure 2:**
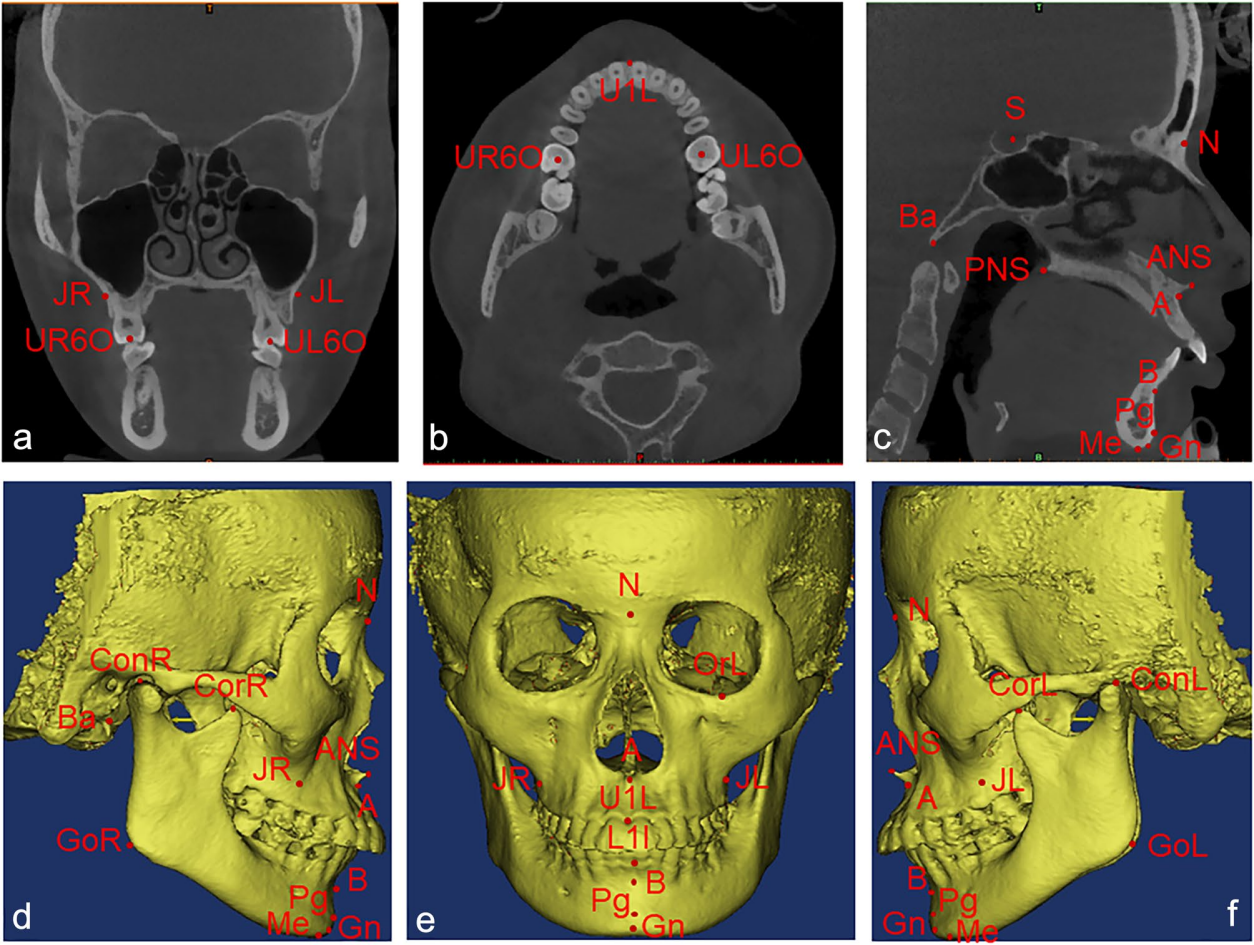
Hard tissue landmarks (**a**) coronal view, (**b**) axial view, (**c**) sagittal view and (**d**–**f**) reconstructed 3D view.

**Figure 3:**
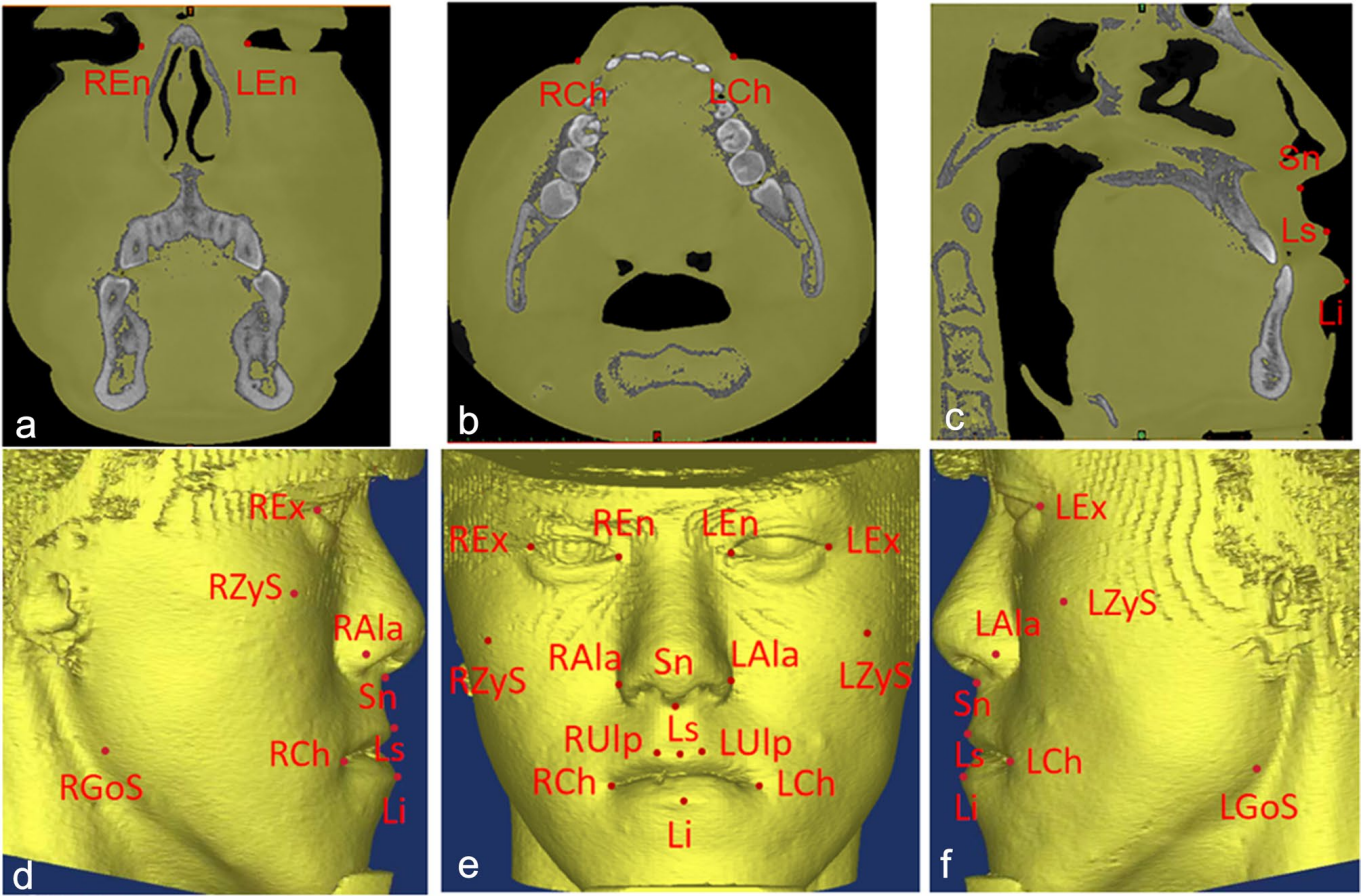
Soft tissue landmarks (**a**) coronal view, (**b**) axial view, (**c**) sagittal view and (**d**–**f**) reconstructed 3D view.

The reference planes (Fig. 1), hard tissue landmarks (Fig. 2), and soft tissue landmarks (Fig. 3) used in this study were based on the literature^18,26,30–32^ and summarized in Table 1. The method of generating midsagittal plane (MSP) was adopted from the literature^17^. Three anatomic points (sella, nasion, and basion) were defined on the cranial base, and the plane that passed through these designated points was defined as MSP. The axial plane passed through the sella point and the nasion point and was perpendicular to MSP; the coronal plane passed through the cranial base point and was perpendicular to MSP and the axial plane respectively (Fig. 1).

**Table 1:** Landmarks and reference planes used in the study.

Landmarks/planes	Abbreviation	Definition
Anatomic porion	Po	Most superior point of the external acoustic meatus
Orbitale	Or	Most inferior point of the infraorbital margin
Anterior nasal spine	ANS	Point located at the tip of the anterior nasal spine
Posterior nasal spine	PNS	The most posterior point of the maxillary posterior nasal spine
Basion	Ba	Middle point on the anterior rim of the occipital foramen
Sella	S	Point in the center of the sella turcica
Nasion	N	Most anterior and median point of the frontonasal suture
Subspinale	A	Point located at the largest concavity of the anterior portion of the maxilla
Supramentale	B	Point located at the largest concavity of the anterior portion of the mental symphysis
Menton	Me	The lowest point of the jaw at the level of the midsagittal plane of the symphysis
Pogonion	Pg	Most anterior point of the bony chin in the median plane
Gnathion	Gn	Most anterior inferior point of the contour of the bony menton
Cusp point of mandibular canine	LMC	Cusp points of bilateral mandibular canines
Maxillary 6 occlusal fossa	U6O	The occlusal fossa of the maxillary first molar
Jugale	J	Point in the intersection of the contour of the maxillary tuberosity with the zygomatic pillar
U1L	U1L	The lowest point of the alveolar bone between the maxillary central incisors
L1I	L1I	The high point of the alveolar bone between the mandibular central incisors
Zygomatic	Zy	The most protruding point of the zygomatic arch
Capitulare	Cap	Point in the center of the head (condyle) of the mandible
Gonion	Go	Most inferior and posterior point on the contour of the gonial angle
Condylion	Con	Most superior and posterior point of the mandibular condyle
Coronoid process	Cor	Upper end of the coronoid process
Frankfort Plane	Frankfort	Plane passing through the right and left anatomic porion points and the left orbitale point (PoR, PoL—OrL)
Midsagittal Plane	MSP	Plane passing through the Sella point (S), the Nasion point (N), the Basion point (Ba)
Horizontal Plane	Horizontal	Plane passing through the Sella point (S), the Nasion point (N) and perpendicular to the midsagittal plane (MSP)
Coronal Plane	Coronal	Plane passing through the base of the Basion point (Ba) and perpendicular to the midsagittal plane (MSP) and the horizontal plane, respectively
Cheilion	Ch	Lateral extent of labial commissure
Labrale superius	Ls	Midpoint of upper vermilion line
Labrale inferius	Li	Most prominent point of lower lip
Subnasale	Sn	Most retruded point in the concavity between nose and upper lip
Endocanthion	En	The soft tissue point located at the inner commissure of each eye fissure
Nasal alare	Ala	Most lateral point of alar contour
Exocanthion	Ex	Outer commisure of palpebral fissure
Soft tissue Zygion	ZyS	Most prominent point on the cheek area
Upper lip point	Ulp	Highest point of upper vermilion
Soft tissue Gonion	GoS	Most lateral point on the mandibular angle close to bony gonion

The Asymmetry Index (AI) was used to evaluate facial asymmetry based on the literature^30^ the formula was: $\sqrt{{\left(Rdx-Ldx\right)}^{2}+{\left(Rdy-Ldy\right)}^{2}+{\left(Rdz-Ldz\right)}^{2}}$, where the R indicates right and L indicates left. The distances between each anatomical point and the three planes were measured with the Mimics 19.0 software and defined as dx, dy, and dz. Variable dx represents distance from anatomical point to MSP, dy represents distance from anatomical point to the coronal plane, and dz represents distance from anatomical point to the axial plane. For solitary paramedial points, dx was used to summarize AI index; for bilateral landmarks, the aforementioned formula was used to calculate AI index.

The intra-rater reliability analysis was performed using 10% of the total samples randomly at one-month intervals. The intraclass correlation coefficient (ICC) was 0.90–0.95, indicating an excellent repeatability.

### Statistical analysis

SPSS software (version 18.0; IBM, Armonk, NY) was used for statistical analysis. The Pearson's chi-square test, One-way ANOVA, and Kruskal–Wallis test were used for statistical comparisons; the Spearman correlation analysis was used to analyze the correlation between the soft tissues and the underlying hard tissues, as well as the facial asymmetry index of Me and ANS point.

## Results

Characteristics of the subjects (gender and age) were similar among the three groups (Table 2).

**Table 2:** Characteristics of the sample.

	Class I (n = 80)	Class II (n = 75)	Class III (n = 66)	Total (n = 221)
Sex (N, %)				
Male	33 (41.3%)	33 (44.0%)	33 (50.0%)	99 (44.8%)
Female	47 (58.7%)	42 (56.0%)	33 (50.0%)	122 (55.2%)
Age (years)				
Mean ± Standard deviation	23.55 ± 3.98	23.67 ± 5.06	22.26 ± 3.97	23.20 ± 4.40
Minimum–Maximum	18–36	18–40	18–34	18–40
ANB (degrees)				
Mean ± Standard deviation	2.73 ± 1.51	7.09 ± 1.61	− 3.61 ± 3.11	2.32 ± 4.79
Minimum–Maximum	0.02–4.95	5.00–11.65	− 13.22 to –0.15	− 13.22 to 11.65

The asymmetry index (AI) values of hard tissues (Table 3) and soft tissues (Table 4) were greater than 0 for all participants. The AI values of hard tissue landmarks (LMC, A, ANS, B, Gn, Me, and Pg) (Table 3) and soft tissue landmark (Li) (Table 4) had significant differences among the skeletal Class I, II, and III groups; these landmarks were generally at, or close to, the midsagittal plane. The hard tissue landmarks Cor and L1I (Table 3) and soft tissue markers Ala and Ch (Table 4) had significant difference between the skeletal Class II and Class III groups.

**Table 3:** Comparison of hard tissue facial asymmetry index among the skeletal Class I, II and III. Data represent median (interquartile range).

Landmarks	Class I	Class II	Class III	P-values
Capitulare (Cap)	6.05 (4.32, 9.97)	6.12 (3.83, 8.67)	5.99 (4.40, 8.80)	0.51
Condylion (Con)	6.13 (4.33, 9.82)	6.46 (4.17, 8.55)	6.17 (4.56, 8.69)	0.84
Coronoid process (Cor)	5.77 (3.53, 9.40)	5.22 (3.36, 7.31)*^b^	6.50 (4.81, 9.29)*^b^	0.11
Gonion (Go)	7.62 (5.38, 13.38)	6.63 (4.92, 10.48)	6.84 (4.70, 10.12)	0.19
Jugale (J)	5.93 (3.14, 9.91)	3.90 (2.82, 6.77)	4.83 (3.07, 9.05)	0.17
Cusp point of mandibular canine (LMC)	6.05 (2.82, 11.93)	4.76 (2.64, 9.73)**	7.91 (4.21, 11.98)**	0.01*
Maxillary 6 occlusal fossa (U6O)	4.74 (3.25, 9.13)	3.82 (2.40, 7.44)	5.07 (3.49, 7.82)	0.18
Zygomatic (Zy)	6.79 (4.27, 10.53)	6.19 (4.01, 9.06)	6.24 (4.30, 9.79)	0.69
Subspinale (A)	1.72 (0.85, 4.07)*^a^	1.10 (0.42, 2.52)*^a^ *^b^	2.18 (0.86, 3.25)*^b^	0.04*
Anterior nasal spine (ANS)	1.52 (0.74, 3.86)	1.17 (0.33, 2.66)*	2.26 (0.94, 3.26)*	0.04*
Supramentale (B)	2.82 (1.08, 5.34)	1.94 (0.53, 5.34)**	4.21 (1.66, 6.82)**	0.02*
Posterior nasal spine (PNS)	1.07 (0.35, 2.41)	0.78 (0.30, 1.86)	1.36 (0.63, 2.26)	0.16
Gnathion (Gn)	2.92 (1.33, 7.44)	2.00 (0.66, 5.82)**	4.70 (1.93, 8.12)**	0.02*
Menton (Me)	2.98 (1.36, 7.34)	2.09 (0.65, 5.74)**	4.78 (1.83, 8.16)**	0.01*
Pogonion (Pg)	2.74 (1.38, 7.38)*^a^	2.09 (0.65, 5.85)*^a^**^b^	4.58 (1.95, 8.07)**^b^	0.01*
U1L (U1L)	2.28 (0.88, 4.87)	1.81 (0.74, 3.34)	2.76 (1.18, 4.17)	0.50
L1I (L1I)	2.89 (0.98, 6.66)	1.78 (0.81, 4.83)*^b^	3.41 (1.54, 5.79)*^b^	0.06

*P < 0.05; **P < 0.01.

^a^Statistical difference between skeletal Class I and II (P < 0.05).

^b^Statistical difference between skeletal Class II and III (P < 0.05).

Bold font indicates statistical difference among the three groups (P < 0.05).

**Table 4:** Comparison of soft tissue facial asymmetry index among the Skeletal Class I, II and III participants. Data represent median (interquartile range).

Landmarks	Class I	Class II	Class III	P-values
Nasal alare (Ala)	4.31 (2.43, 8.70)	3.74 (2.53, 6.07)*^b^	4.70 (3.17, 7.34)*^b^	0.13
Cheilion (Ch)	5.96 (3.33, 11.77)	4.59 (2.70, 8.76)*^b^	7.05 (3.63, 10.76)*^b^	0.08
Endocanthion (En)	2.80 (1.76, 4.79)	2.97 (1.70, 4.34)	3.06 (2.25, 4.74)	0.31
Labrale superius (Ls)	2.41 (1.14, 6.16)	1.96 (0.97, 3.83)	2.78 (1.39, 4.76)	0.22
Labrale inferius (Li)	3.03 (1.25, 7.11)*^a^	1.85 (0.52, 4.26)*^a^*^b^	3.30 (1.35, 5.91)*^b^	0.03*
Subnasale (Sn)	2.29 (0.65, 4.79)	1.52 (0.67, 3.20)	2.45 (1.34, 4.14)	0.18
Exocanthion (Ex)	3.48 (5.81, 9.88)	4.23 (5.42, 8.56)	4.52 (6.71, 8.61)	0.67
Soft tissue Gonion (GoS)	5.91 (8.90, 15.89)	4.62 (7.19, 10.56)	6.04 (7.50, 11.2)	0.08
Soft tissue Zygion (ZyS)	2.16 (5.58, 12.22)	1.41 (4.39, 7.23)	2.30 (5.99, 9.51)	0.16
Upper lip point (Ulp)	4.15 (7.46, 13.11)	4.20 (6.23, 9.37)	4.91 (7.20, 9.80)	0.53

*P < 0.05; **P < 0.01.

^a^Statistical difference between skeletal Class I and II (P < 0.05).

^b^Statistical difference between skeletal Class II and III (P < 0.05).

Bold fonts indicate statistical difference among the three groups (**P < 0.05**).

**Figure 4:**
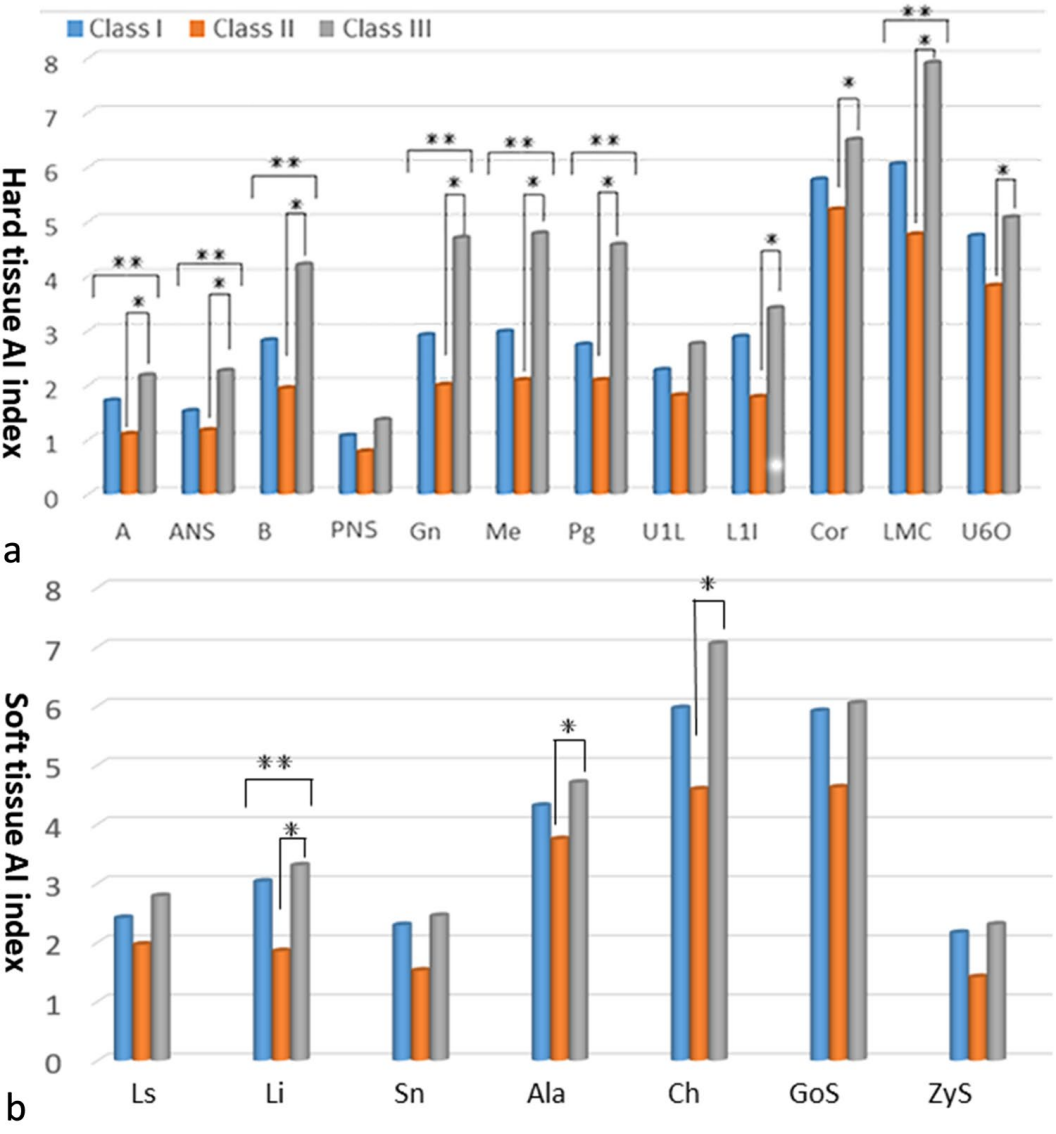
Comparison of the asymmetry index (AI) among the Class I, II, and III. (**a**) The hard tissue asymmetry: Class III > I > II. (**b**) The soft tissue asymmetry: Class III > I > II. *Statistical difference between the two groups, *P* < 0.05. **Statistical difference among the three groups, *P* < 0.05.

The skeletal Class III group had the greatest facial asymmetry for most hard tissue and some soft tissue landmarks (i.e. A, ANS, B, PNS, Gn, Me, Pg, U1l, L1l, Ala, Ch, Ls, Li, Sn, Cor, Lmc, and U6O), followed by the skeletal Class I, and skeletal Class II (Tables 3 and 4, Fig. 4); and these landmarks were at, or close to, the midsagittal plane. The AI values of soft and hard tissue landmarks generally increased from the top to the bottom of the face, including ANS, A, U1L, L1I, B, Pg, Gn, Me, En, ZyS, Ala, Ch, GoS, Ls, Li (Tables 3 and 4, Fig. 4).

**Figure 5:**
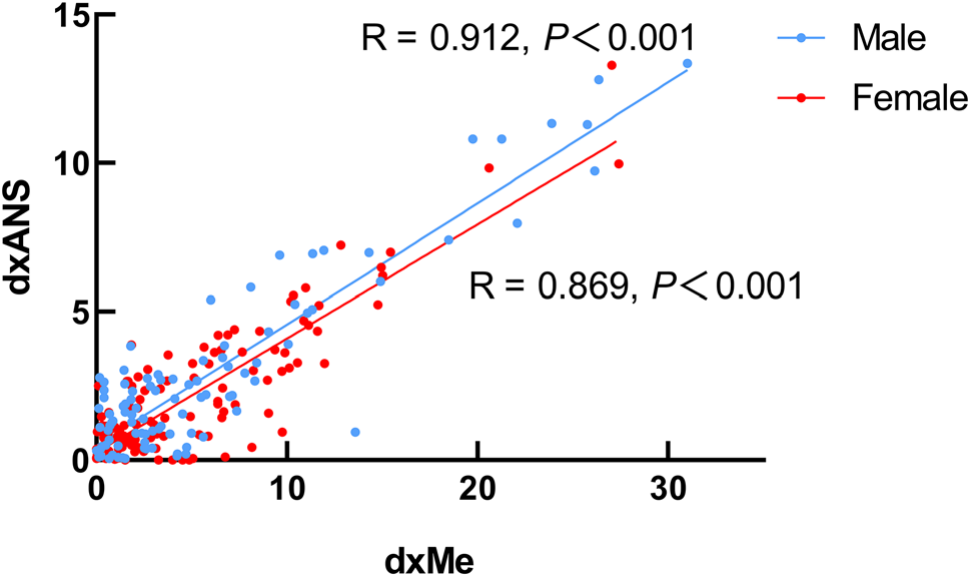
The correlation coefficients (P-values) of AI index of ANS and Me.

**Figure 6:**
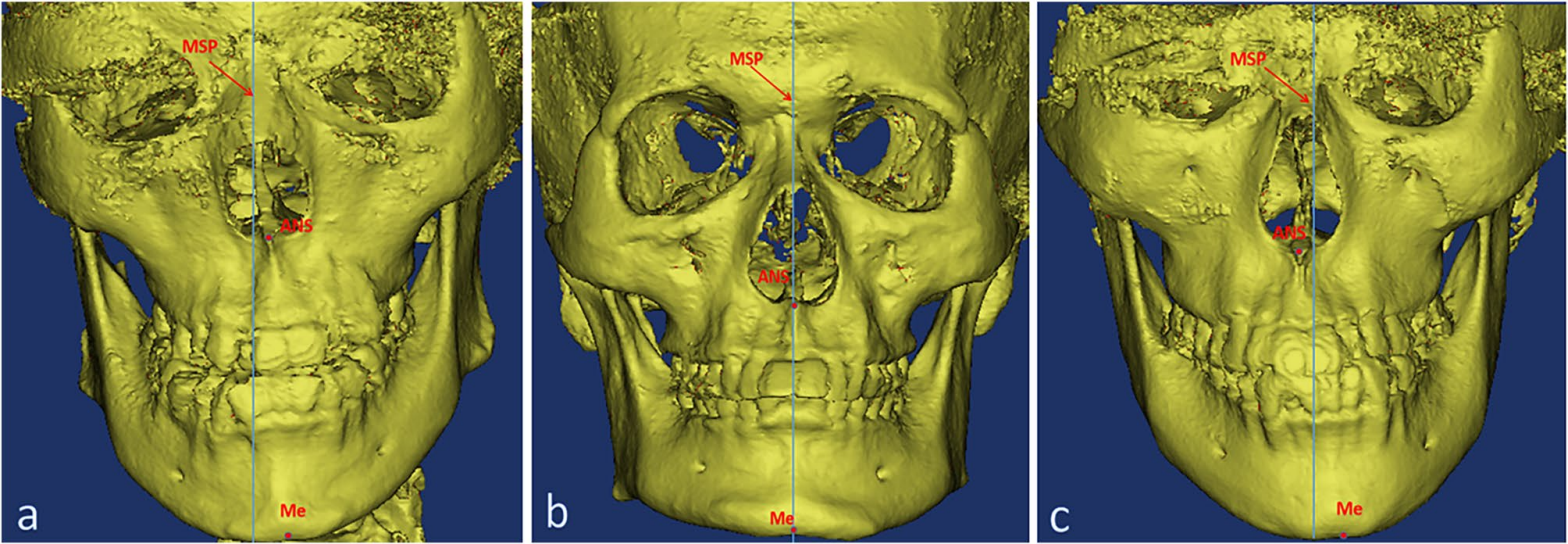
Deviation direction of ANS and Me. (**a**) Deviation directions consistent (**b**) ANS and Me at the MSP (**c**) Deviation directions inconsistent.

There were positive correlations (r_s_ = 0.71—0.87) between six soft tissue landmarks (Ch, Sn, Ls, Li, GoS and ZyS) and their corresponding hard tissue landmarks (LMC, A, U1L, L1I, Go and Zy) (P < 0.01 for all) (Table 5). A high AI value correlation was also found between the hard tissue landmark ANS and Me (Fig. 5), however 21.3% of patients had ANS and ME point deviation direction inconsistency (Table 6 and Fig. 6). There was no significant difference of the deviation direction of these two points between male and female subjects (Table 6).

**Table 5:** The spear correlation coefficients (P-values) of facial asymmetry index between the soft tissues and hard tissues in skeletal Class I, II, and III subjects.

	Cheilion (Ch)	Subnasale (Sn)	Labrale superius (Ls)	Labrale inferius (Li)	Soft tissue Gonion (GoS)	Soft tissue Zygion (ZyS)
Cusp point of mandibular canine (LMC)	0.87 (P < 0.001)					
Subspinale (A)		0.85 (P < 0.001)				
U1L (U1L)			0.86 (P < 0.001)			
L1I (L1I)				0.84 (P < 0.001)		
Go					0.85 (P < 0.001)	
Zy						0.78 (P < 0.001)
Skeletal Class II						
Cusp point of mandibular canine (LMC)	0.77 (P < 0.001)					
Subspinale (A)		0.78 (P < 0.001)				
U1L (U1L)			0.78 (P < 0.001)			
L1I (L1I)				0.81 (P < 0.001)		
Go					0.79 (P < 0.001)	
Zy						0.71 (P < 0.001)
Skeletal Class III						
Cusp point of mandibular canine (LMC)	0.79 (P < 0.001)					
Subspinale (A)		0.84 (P < 0.001)				
U1L (U1L)			0.81 (P < 0.001)			
L1I (L1I)				0.83 (P < 0.001)		
Go					0.81 (P < 0.001)	
Zy						0.82 (P < 0.001)

A positive rs value indicates a positive correlation. The absolute value of rs is 0.8–1.0, indicating an extremely strong correlation between variables; 0.6–0.8 indicates a strong correlation between variables.

**Table 6:** No statistical significant difference of ANS, Me, and deviation direction between the male and female participants (n, %).

	Male (n = 99)	Female (n = 122)	Total (n = 221)	χ^2^	*P-value*
ANS					
Right	47 (47.5%)	55 (45.1%)	102 (46.2%)	5.049	0.081
Left	52 (52.5%)	61 (50.0%)	113 (51.1%)		
Middle	0 (0%)	6 (4.9%)	6 (2.7%)		
Me					
Right	45 (45.5%)	50 (41.0%)	95 (43%)	0.924	0.656
Left	53 (53.5%)	69 (56.6%)	122 (55.2%)		
Middle	1 (1.0%)	3 (2.5%)	4 (1.8%)		
Deviation direction					
Consistent	75 (75.8%)	99 (81.1%)	174 (78.7%)	0.948	0.330
Inconsistent	24 (24.2%)	23 (18.9%)	47 (21.3%)

## Discussion

A number of clinical studies have evaluated facial asymmetry from the aspects of hard tissues^8,10,18,22,23^ soft tissues^26,33^ and both^9,17,25^. The findings of this study suggest that the skeletal Class II patients showed relatively more facial symmetry than skeletal Class III patients and the soft tissues showed a similar asymmetry as the underlying hard tissues.

Among all landmarks measured in the present study, 12 hard tissue landmarks and 6 soft tissue landmarks showed that the skeletal Class III asymmetry index was the largest and the skeletal Class II asymmetry index was the smallest, but there was no statistical significance between Class I and III, nor between Class I and II. This is consistent with previous studies^18,22^. A large population based study in Japan (n = 1800) found that the patients showing no chin deviation was 36.9%, 41.4%, and 33.6% in skeletal Class I, II and III malocclusions, respectively^5^. It has been found that skeletal Class II patients are likely to have relatively less mandibular growth than skeletal Class III patients who generally exhibit greater mandibular growth and may be more likely to be affected by postnatal environmental influence during the relatively longer jaw growth period^34^. A number of studies have found that Class III patients had more facial asymmetry^12,17,23^, which may be due to the environmental influences and habitual chewing on one side^5^.

It has been found that the chin deviation to the left (70–90%) was more prevalent than the deviation to the right^19,22^. In the current study, however, no significant difference of the chin (ME point) deviation was found between the left and right. This may be due to the differences in study population and evaluation methods.

The median-sagittal plane (MSP) is often used as a reference for the mirroring and segmentation processes to assess facial symmetry and asymmetry. The current study adopted the method described in the literature^17^. A number of methods can be used to generate the midplane^35–38^. The cephalometric landmark based midplane is the traditional approach; it is relatively easy but sometimes the unpaired landmarks are not exactly in the middle of the face, resulting in an unreliable MSP^39^. Morphometric measurement has been reported to define the true MSP, but very few software programs have this function^36^. A study compared two digital methods (interactive closest point algorithm (ICP) and Procrustes analysis (PA)) to determine the median sagittal plane. Results showed that ICP and PA were similar for subjects with no obvious facial asymmetry, but for the subjects with obvious facial asymmetry, it remained unclear which one computes the ideal MSP^37^. To date, the best approach to define the true MSP is still contested in the literature.

Although facial asymmetries are mostly perceived on horizontal and transversal planes by patients and observers, it can occur in all three planes of space^40^. The current study analyzed the facial asymmetry in three dimensions. In the literature, facial asymmetry has been evaluated morphologically and classified into different categories^41–44^. In addition to morphological asymmetry, there can be spatial asymmetry. It has been found that some subjects presented no difference in the size and length between the right and left mandibular sides but still had Me point and lower apical base midline deviated to one side. This type of asymmetry has also been described as functional asymmetry^44^, and was suggested to be intercepted at an early stage because the condyle and fossa could adapt easily to the deviated mandibular position. It is clinically important to qualify and quantify the dental, skeletal, soft tissue, and functional asymmetry for the accurate diagnosis and appropriate treatment.

The external (soft tissues) and internal (hard tissues) appearances of facial asymmetry are both clinically important, and they are often correlated. It has been found that there was a high covariance between the soft tissues and the underlying hard tissues^25^. A CBCT study evaluated the right and left difference of facial soft tissue landmarks in subjects with normal occlusion and found that the bilateral landmarks presented greater asymmetry and higher variability than the midline landmarks^26^.

It is interesting to note that in the current study, although there was a high correlation between the asymmetry index of ANS and ME points, these two landmarks deviated to different sides in about 20% patients. There is evidence showing that the deviations at the tip of the nose were always rated as more asymmetric than the same deviations of the chin; the nasal tip shift left-sided and chin shift right-sided were judged as significantly more asymmetric^45^. Whether the deviation direction of ANS and ME exacerbates or alleviates facial asymmetry still requires further studies.

There are limitations in the present study. The reproducibility of soft tissue landmarks is generally low^46^. The eight soft tissue landmarks used in the current study were relatively easy to locate, however, most of them are at, or close to, the midface, which may not be adequate to reflect whole facial asymmetry. Future studies using morphometric methodology, 3D stereophotogrammetry, and dynamic motion capture may provide a better understanding of the facial asymmetry, by taking into consideration of the shape, size, and volume of the facial soft and hard structures.

## Conclusions

All patients presented facial asymmetry to a different degree. Skeletal Class III subjects presented greater facial asymmetry than skeletal Class II patients. Midface soft tissues showed a similar asymmetry as the underlying hard tissues rather than a compensation of the hard tissue asymmetry. The inconsistency in the deviation of Me and ANS may exacerbate facial asymmetry.

## Data Availability

The datasets generated during and/or analyzed during the current study are available from the corresponding author on reasonable request.

## References

[1] Cheong YW, Lo LJ (2011). Facial asymmetry: Etiology, evaluation, and management. Chang Gung Med. J..

[2] Sheats RD, McGorray SP, Musmar Q, Wheeler TT, King GJ (1998). Prevalence of orthodontic asymmetries. Semin. Orthod..

[3] Thiesen G, Gribel BF, Freitas MPM, Oliver DR, Kim KB (2018). Mandibular asymmetries and associated factors in orthodontic and orthognathic surgery patients. Angle Orthod..

[4] Ramirez-Yañez GO, Stewart A, Franken E, Campos K (2011). Prevalence of mandibular asymmetries in growing patients. Eur. J. Orthod..

[5] Haraguchi S, Iguchi Y, Takada K (2008). Asymmetry of the face in orthodontic patients. Angle Orthod..

[6] Thiesen G, Gribel BF, Freitas MP (2015). Facial asymmetry: A current review. Dental. Press J. Orthod..

[7] Solem RC (2016). Congenital and acquired mandibular asymmetry: Mapping growth and remodeling in 3 dimensions. Am. J. Orthod. Dentofacial. Orthop..

[8] Thiesen G, Gribel BF, Freitas MPM, Oliver DR, Kim KB (2017). Craniofacial features affecting mandibular asymmetries in skeletal Class II patients. J. Orofac. Orthop..

[9] Chen YJ (2019). Characterization of facial asymmetry in skeletal Class III malocclusion and its implications for treatment. Int. J. Oral. Maxillofac. Surg..

[10] Lv W, Nie Q, Gu Y (2021). Three-dimensional analysis of mandibular characteristics in patients with skeletal Class II malocclusion and chin deviation. Am. J. Orthod. Dentofacial. Orthop..

[11] Thiesen G, Freitas MPM, Araújo EA, Gribel BF, Kim KB (2018). Three-dimensional evaluation of craniofacial characteristics related to mandibular asymmetries in skeletal Class I patients. Am. J. Orthod. Dentofacial. Orthop..

[12] Teng C, Liu C, Yu Q, Liu S (2021). Cone-beam Computed Tomography-based three-dimensional cephalometric analysis of mandible symmetry and the occlusal plane of adult patients with high-angle skeletal class III malocclusion and jaw deformity. Arch Oral. Biol..

[13] Lee H (2012). Mandibular dimensions of subjects with asymmetric skeletal class III malocclusion and normal occlusion compared with cone-beam computed tomography. Am. J. Orthod. Dentofacial. Orthop..

[14] Park JU, Kook YA, Kim Y (2012). Assessment of asymmetry in a normal occlusion sample and asymmetric patients with three-dimensional cone beam computed tomography: A study for a transverse reference plane. Angle Orthod..

[15] Kim HO, Lee W, Kook YA, Kim Y (2013). Comparison of the condyle-fossa relationship between skeletal class III malocclusion patients with and without asymmetry: A retrospective three-dimensional cone-beam computed tomography study. Korean J. Orthod..

[16] Tyan S (2016). Three-dimensional analysis of molar compensation in patients with facial asymmetry and mandibular prognathism. Angle Orthod..

[17] Duran GS, Dindaroglu F, Kutlu P (2019). Hard- and soft-tissue symmetry comparison in patients with Class III malocclusion. Am. J. Orthod. Dentofacial. Orthop..

[18] Sievers MM, Larson BE, Gaillard PR, Wey A (2012). Asymmetry assessment using cone beam CT. A Class I and Class II patient comparison. Angle Orthod..

[19] Kim EJ (2011). Maxillofacial characteristics affecting chin deviation between mandibular retrusion and prognathism patients. Angle Orthod..

[20] Thiesen G, Freitas MPM, Gribel BF, Kim KB (2019). Comparison of maxillomandibular asymmetries in adult patients presenting different sagittal jaw relationships. Dental. Press J. Orthod..

[21] Zhao J, Xu Y, Wang J, Lu Z, Qi K (2023). 3-dimensional analysis of hard- and soft-tissue symmetry in a Chinese population. BMC Oral. Health..

[22] Severt TR, Proffit WR (1997). The prevalence of facial asymmetry in the dentofacial deformities population at the University of North Carolina. Int. J. Adult Orthodon. Orthognath. Surg..

[23] Fong JH (2010). Analysis of facial skeletal characteristics in patients with chin deviation. J. Chin. Med. Assoc..

[24] Ackerman JL, Proffit WR, Sarver DM (1999). The emerging soft tissue paradigm in orthodontic diagnosis and treatment planning. Clin. Orthod. Res..

[25] Young NM (2016). Facial surface morphology predicts variation in internal skeletal shape. Am. J. Orthod. Dentofacial. Orthop..

[26] Hwang HS (2012). Three-dimensional soft tissue analysis for the evaluation of facial asymmetry in normal occlusion individuals. Korean J. Orthod..

[27] Mendoza LV (2018). Linear and volumetric mandibular asymmetries in adult patients with different skeletal classes and vertical patterns: A cone-beam computed tomography study. Sci Rep..

[28] Fu, M. K*. Orthodontics Course*. 2nd ed. (People’s Health Publishing House, 2010).

[29] Hussels W, Nanda RS (1984). Analysis of factors affecting angle ANB. Am. J. Orthod..

[30] Katsumata A (2005). 3D-CT evaluation of facial asymmetry. Oral Surg. Oral Med. Oral Pathol. Oral Radiol. Endod..

[31] Hwang HS, Hwang CH, Lee KH, Kang BC (2006). Maxillofacial 3-dimensional image analysis for the diagnosis of facial asymmetry. Am. J. Orthod. Dentofacial. Orthop..

[32] Maeda M (2006). 3D-CT evaluation of facial asymmetry in patients with maxillofacial deformities. Oral Surg. Oral Med. Oral Pathol. Oral Radiol. Endod..

[33] Xue Z (2020). Three-dimensional dynamic analysis of the facial movement symmetry of skeletal class iii patients with facial asymmetry. J. Oral Maxillofac. Surg..

[34] Xiong X (2020). Distribution of various maxilla-mandibular positions and cephalometric comparison in Chinese skeletal class II malocclusions. J. Contemp. Dent. Pract..

[35] Noh HK, Kim HJ, Park HS (2023). Differences in positions of cone-beam computed tomography landmarks in patients with skeletal Class III facial asymmetry according to midsagittal planes. Korean J. Orthod..

[36] Dobai A (2018). Landmark-based midsagittal plane analysis in patients with facial symmetry and asymmetry based on CBCT analysis tomography. J. Orofac. Orthop..

[37] Xiong Y, Zhao Y, Yang H, Sun Y, Wang Y (2016). Comparison between interactive closest point and procrustes analysis for determining the median sagittal plane of three-dimensional facial data. J. Craniofac. Surg..

[38] Shin SM (2016). Statistical shape analysis-based determination of optimal midsagittal reference plane for evaluation of facial asymmetry. Am. J. Orthod. Dentofacial. Orthop..

[39] Damstra J, Fourie Z, De Wit M, Ren Y (2012). A three-dimensional comparison of a morphometric and conventional cephalometric midsagittal planes for craniofacial asymmetry. Clin. Oral. Investig..

[40] Jackson TH (2013). Face symmetry assessment abilities: Clinical implications for diagnosing asymmetry. Am. J. Orthod. Dentofacial. Orthop..

[41] Leung MY, Leung YY (2018). Three-dimensional evaluation of mandibular asymmetry: A new classification and three-dimensional cephalometric analysis. Int. J. Oral Maxillofac. Surg..

[42] Baek C, Paeng JY, Lee JS, Hong J (2012). Morphologic evaluation and classification of facial asymmetry using 3-dimensional computed tomography. J. Oral Maxillofac. Surg..

[43] Schmid W, Mongini F, Felisio A (1991). A computer-based assessment of structural and displacement asymmetries of the mandible. Am. J. Orthod. Dentofacial. Orthop..

[44] Joondeph DR (2000). Mysteries of asymmetries. Am. J. Orthod. Dentofacial. Orthop..

[45] Meyer-Marcotty P, Stellzig-Eisenhauer A, Bareis U, Hartmann J, Kochel J (2011). Three-dimensional perception of facial asymmetry. Eur. J. Orthod..

[46] Gwilliam JR, Cunningham SJ, Hutton T (2006). Reproducibility of soft tissue landmarks on three-dimensional facial scans. Eur. J. Orthod..

